# Pre-clinical evaluation of a divalent liposomal vaccine to control invasive candidiasis

**DOI:** 10.1038/s41541-025-01183-0

**Published:** 2025-06-13

**Authors:** Augusto Costa-Barbosa, Maria Inês Pacheco, Andreia C. Gomes, Tony Collins, Manuel Vilanova, Célia Pais, Alexandra Correia, Paula Sampaio

**Affiliations:** 1 Centre of Molecular and Environmental Biology (CBMA) /Aquatic Research Network (ARNET) Associate Laboratory, Universidade do Minho, Campus de Gualtar, Braga, Portugal; 2 Institute of Science and Innovation for Sustainability (IB-S), Universidade do Minho, Campus de Gualtar, Braga, Portugal; 3 i3S, Instituto de Investigação e Inovação em Saúde, Universidade do Porto, Porto, Portugal; 4 IBMC, Instituto de Biologia Molecular e Celular, Universidade do Porto, Porto, Portugal

**Keywords:** Microbiology, Vaccines

## Abstract

*Candida albicans* causes systemic infections with 20–50% mortality in critically ill and immunocompromised patients, despite antifungal treatment. Current therapies face limitations, including toxicity and resistance, underscoring the need for prophylactic vaccines. This study presents a novel divalent liposomal vaccine, delivering *C. albicans* Cht3 and Sap2 antigens. Vaccination induced protective Th1/Th17 immunity, a balanced Th1/Th2 ratio, antigen-specific antibodies, and boosted macrophage activity, improving survival in a mouse model of invasive candidiasis.

*Candida species* are common yeast in the human microbiota but can become invasive in critical care settings and among patients with central venous catheters, recent surgical procedures, or those receiving total parenteral nutrition, organ transplants, chemotherapy, broad-spectrum antibiotics, or hemodialysis^1^. Access to diagnostics is high, but evidence-based treatments remain limited. This challenge is worsened by the difficulty in diagnosing certain forms, such as abdominal candidiasis, where blood cultures have a positivity rate of <15%^1^. The Centers for Disease Control and Prevention (CDC) reports ~25,000 annual candidemia cases in the United States (~68 daily) while in Europe, the incidence is estimated at around 79 cases per day^2^. Limited effective antifungal therapies and rising of antifungal resistance contributes to high mortality rates and hospitalization costs associated with invasive candidiasis^2,3^. In fact, invasive candidiasis has a 20–50% mortality rate, despite antifungal treatment, with prolonged hospital stays (2–8 weeks) and high economic burden.

*Candida albicans* remains the most frequently isolated species from invasive candidiasis although its incidence is declining relative to other species like *C. glabrata*, *C. tropicalis*, *C. parapsilosis*, and *C. krusei*, mainly due to increasing antifungal resistance challenges^4^. Protection against invasive candidiasis relies on cellular immunity, particularly in a balanced Th1/Th17 response that promotes IFN-γ and IL-17-mediated neutrophil activation^5,6^. Monocytes, macrophages and dendritic cells, along with neutrophils, are important phagocytes in invasive candidiasis, enhancing phagocytosis and inflammation through complement activation^5^. B cells, primarily responsible for antibody production, also contribute to antigen presentation and cytokine production. Passive transfer studies indicate that antigen-specific IgG levels are crucial for recovery, promoting opsonic activity and complement activation^7,8^. Trained immunity, induced by epigenetic reprogramming of myeloid progenitors, have also been implicated in protection against a invasive infection by promoting increased TNF-α and IL-6 levels after a secondary stimulation^9^. Preventing invasive candidiasis is challenging due to the lack of a vaccine and plasticity of *C. albicans*. In the clinical trial database (https://clinicaltrials.gov) few peptide-based anti-*Candida* vaccine formulations have progressed to clinical trials. NDV-3A, which uses the *C. albicans* Als3 N-terminus with alhydrogel (NCT01926028), and PEV7, which incorporates a truncated *C. albicans* Sap2 into influenza virosomes (NCT01067131), are the most exploited^10,11^. Additionally, Candi5V, a pentavalent bioconjugate vaccine (NCT06190509), and the autovaccine Vacucis Candida® (NCT05289375) are either in clinical trials or under consideration. However, all proposed vaccines are being developed for efficacy in mucosal infections, such as vulvovaginal or oral candidiasis (Reviewed in ref. ^12^).

Building on our previous findings demonstrating DODAB:MO liposomes as efficient antigen delivery system and adjuvant with crude cell wall surface protein extract (CWSP)^13,14^ and recombinant *C. albicans* chitinase 3 (Cht3)^8,15^, we evaluated a new divalent formulation containing recombinant Cht3 and Sap2. Cht3, involved in chitin remodeling^16^, and Sap2, a secreted protease and key virulence factor^17^, were selected to target distinct pathogenic mechanisms. in *C. albicans*. This study used a mouse model of systemic candidiasis to assess the efficacy of the divalent liposomal vaccine for improved delivery, immunogenicity, and protection against invasive *C. albicans* infection.

In this study we investigated whether a divalent liposomal formulation combining recombinant Cht3 and Sap2 could provide protection against *C. albicans* invasive infection. Cht3 (~150 kDa) and Sap2 (~37 kDa) were expressed, purified, and incorporated into DODAB:MO liposomes (Fig. S1). Liposomes contained 50 µg/mL total protein (5 µg/mL Cht3, 45 µg/mL Sap2), reflecting native chitinase 3 levels and effective doses from our prior work^8,14,15^. Dynamic Light Scattering (DLS) and CryoSEM analyses indicated that the formulation preserved liposome integrity (200–400 nm size, +56.6 ± 0.7 mV ζ-potential) (Fig. S1). Before in vivo validation, in vitro evaluation of the liposomal formulation showed no cytotoxicity after 24–48 h (Table S1), and reduced TNF-α stimulation, confirming safety (Fig. S1).

Mice were immunized following the immunization schedule in Fig. 1a. Mice were immunized as in Fig. 1a, then euthanized and their spleens harvested for analysis. The divalent vaccine elicited strong immunomodulatory effects, significantly enhancing proliferation of total, CD4 + , and CD8+ lymphocytes, with CD4+ cells showing the strongest response, after antigen specific stimulation (Figs. 1b, S2). Cytokine analysis from the culture supernatants of antigen-stimulated splenocytes revealed elevated IL-17A, IFN-γ, and IL-10 levels in splenocytes from vaccinated mice in response to specific antigen (Cht3:Sap2), compared to sham-immunized controls, while IL-4 remained low (Fig. 1c). The divalent vaccine induced a strong Th1-biased response, with a 5.1-fold increase in the IFN-γ/IL-4 ratio compared to the protein-only group (*P* = 0.039). These findings indicate that the divalent formulation enhances memory CD4 + T cell activation and promotes a protective Th1/Th17 protective immune response, with DODAB:MO liposomes serving as effective delivery systems. In this study, three weeks post-immunization, mice receiving liposomal formulations had significantly higher specific IgG levels than controls (Fig. 1d), promoting opsonization and recovery, as previously described^8,14^. Mice immunized with *C. albicans* β-glucan exhibited trained immunity, enhancing cytokine responses such as TNF-α and IL-6 in bone marrow derived macrophages (BMDMs) after in vitro *C. albicans* restimulation. In this study, BMDMs from immunized mice showed significantly higher IL-6, and TNF-α levels after *C. albicans* restimulation than control (Fig. 1e), suggesting that divalent immunization induced a trained immunity cytokine profile in BMDMs, consistent with previous report^9^.

**Fig. 1:**
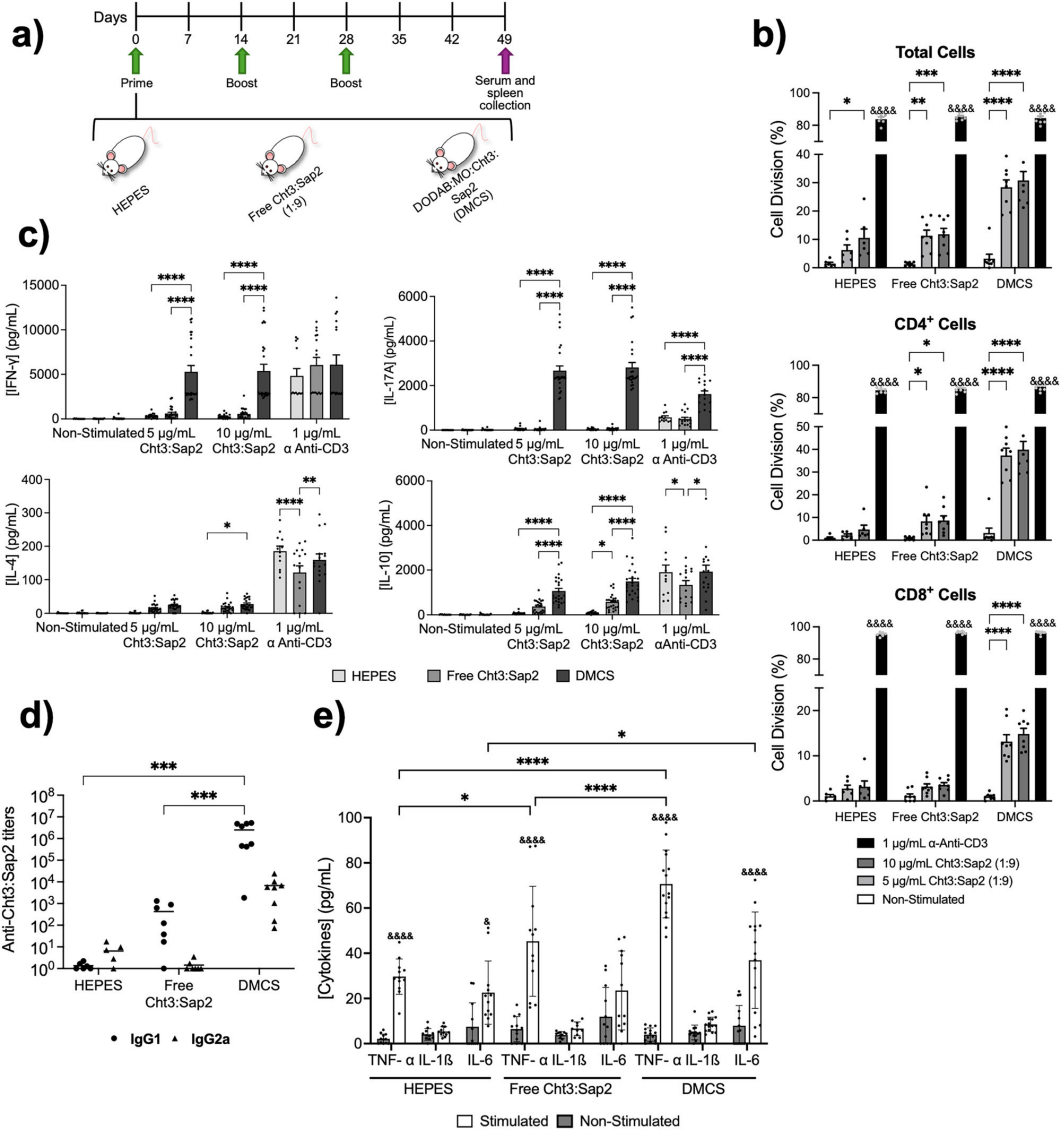
DMCS Induces Th1/Th17 immunity, enhanced antibody response, and trained immunity cytokine profile against *C. albicans.* **a** Immunization schedule. **b** Proliferation of Total Cells, CD4^+^ cells, and CD8^+^ cells, and **c** Levels of IFN-γ, IL-17A, IL-4, and IL-10 from splenocytes of immunized mice after non-stimulation conditions or stimulated with Cht3:Sap2 at 5 or 10 µg/mL or stimulated with anti-CD3 (1 µg/mL). **d** Anti-Cht3:Sap2 IgG1 (●) and IgG2a (▲) responses in serum from mice immunized with HEPES buffer, Free Cht3:Sap2, or DMCS. **e** Quantification of TNF-α, IL-1β, and IL-6 production by BMDMs from immunized mice after 24 h stimulation with *C. albicans* cells. Results are presented as mean ± SEM. Significant differences are indicated by asterisks: *P < 0.05; **P < 0.01; ***P < 0.001; ****P < 0.0001. Differences between the αCD3 group and all other stimulation conditions within each group, or between stimulated and non-stimulated BMDMs, are marked with ampersands above the bars: ^&^*P* < 0.05; ^&^ ^&^ ^&^ ^&^*P* < 0.0001.

Immunized mice were challenged with a lethal dose of a *C. albicans* clinical strain (124 A) following the schedule in Fig. 2a to evaluate the DMCS vaccine’s protective effect. Infection progressed similarly in the HEPES buffer and Empty liposome groups (*P* = 0.5786, Fig. S3), which were combined as the Control group. The DMCS vaccination provided strong protection against infection by a lethal dose of *C. albicans*, significantly extending mice survival (Fig. 2b). While the control group and those receiving free proteins succumbed to infection with a median survival time of 16 days, the divalent formulation markedly improved survival to 29 days (Fig. 2c) and the free rCht3:rSap2 showed no survival increase. Moreover, DMCS-treated mice exhibited the better overall health scores (Fig. 2d, Table S2), and minimal weight loss (Fig. 2e, Table S3). These findings highlight that immunization with DODAB:MO liposomes with Cht3 and Sap2 provides strong protection against lethal *C. albicans* infection likely via robust a Th1/Th17 response, enhanced antigen-specific IgG levels, combined with a trained immunity cytokine profile.

**Fig. 2:**
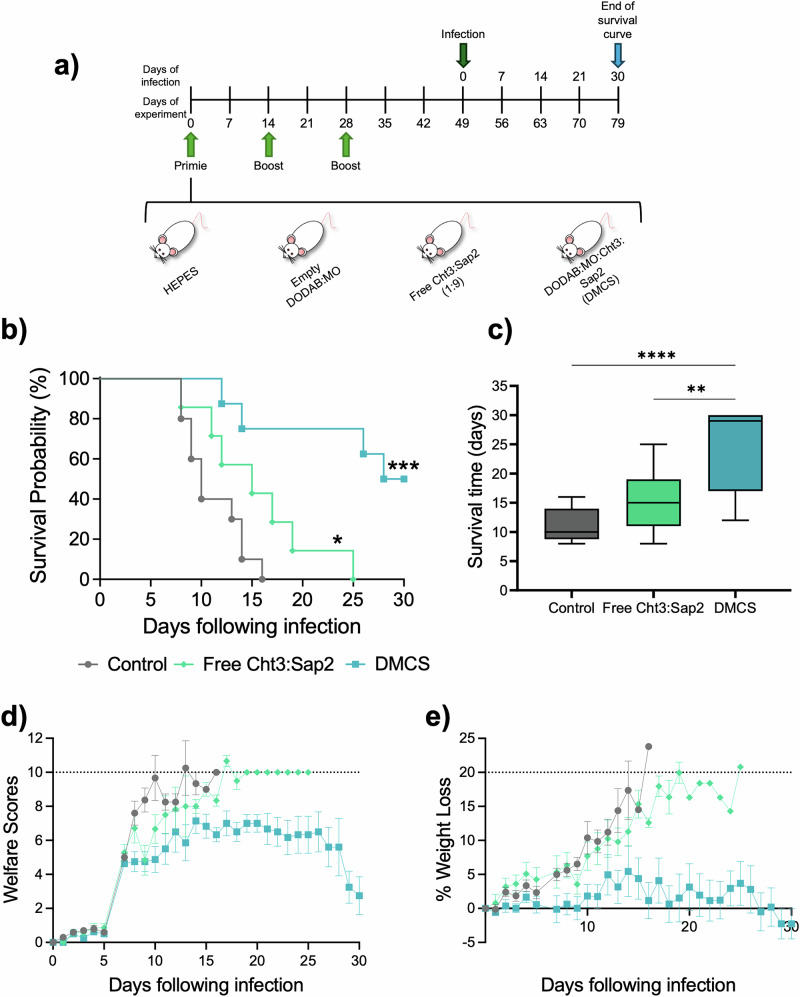
Vaccination with DMCS protects from a lethal *C. albicans* systemic infection. **a** Scheme of BALB/c mice s.c. immunization with either HEPES buffer, empty liposomes, free Cht3:Sap2 (1:9), or DMCS (liposomes with Cht3:Sap2), and i.v. infection with *C. albicans*. Infection progressed similarly in the HEPES buffer and empty liposome groups (*P* = 0.5786), so they were combined in the Control group for clearer comparison and representation. Statistical comparisons between the control and the other immunized groups were performed thought the Log-rank (Mantel-Cox) test. **b** Survival curve for immunized mice after infection with *C. albicans*; **c** Average survival time for the immunization groups following infection, analyzed by one-way ANOVA. **d** Welfare scores for mice following infection; **e** Percentage weight loss of mice following infection. Endpoint values for welfare scores and weight loss are indicated by dashed lines. Significant differences are indicated by * above the lines (*P < 0.05; **P < 0.01; ***P < 0.001; ****P < 0.0001).

*Candida albicans* is usually a harmless commensal, but in immunocompromised individuals, it can cause severe invasive infections. This has encouraged research into prophylactic vaccines, particularly for high-risk patients undergoing immunosuppressive treatments^18^. Different types of *C. albicans* antigens have been used to induce an immune response, from cell wall polysaccharides to several proteins, glycopeptides and peptides, many times with tags associated^19^. Our synthetic anti-*Candida* vaccine approach evolved from a complex mixture of cell wall surface proteins^13,14^ to a study-driven selection of Cht3 and Sap2, expressed recombinantly and used directly for *Candida-*only epitopes. While aluminum salts are well-established adjuvant, their propensity to induce Th2 differentiation make them less suitable for targeting *C. albicans* infections, which require Th1/Th17 responses^12^. Following the COVID-19 pandemic, liposome-based platforms with cationic lipids like ALC-0315 (Pfizer) and SM-102 (Moderna), or other as synthetic monophosphoryl lipid and QS-21 have gained popularity.

Liposomes, with lipid bilayers, can carry both hydrophilic and hydrophobic molecules, enhancing immune responses by targeting immune cells and promoting antigen cross-presentation. While alternatives like cubosomes and polymeric nanocapsules exist, liposomes remain a clinically validated platform, balancing efficacy, safety, and versatility^20^. DODAB:MO liposomes act as both an antigen delivery system and adjuvant in a single-component platform. Thus, this study builds on previous work and describes a divalent protein-based DODAB:MO liposomal vaccine with recombinant Cht3 and Sap2 to broaden the immunity, overcome fungal immune evasion, and enhance protection against invasive infection, without additional adjuvants.

Intradermal delivery efficiency depends on particle size, with smaller particles (<50 nm) clearing rapidly and larger particles (>200 nm) showing limited tissue penetration but favoring uptake by antigen-presenting cells (APCs). This liposomal formulation (~450 nm size, positive ζ-potential) preserved the structure and physicochemical properties, supporting APC-mediated lymph node targeting and immune activation^8,13^, while avoiding high antigen doses and nonspecific endocytosis clearance^14,21^. Immunization with the divalent formulation increased specific CD4 + T cells, especially those producing IFN-γ and IL-17A, as in other vaccine-oriented studies^7,10,19^. Cytokine analysis of splenocyte cultures revealed increased IFN-γ, IL-17, and IL-10 production, reinforcing a Th1/Th17 response for effective neutrophil activation^5,6^. The vaccine oriented studies using agglutinin-like sequences (Als) 3 highlighted the importance of the Th1/Th17 cell-mediated response in providing protection against systemic candidiasis^10^. Immunization with DMCS induces high titers of anti-Cht3:Sap2 antibodies, compared to proteins alone. Although the role of humoral responses in systemic candidiasis is controversial, several studies highlight the need for inducing protective humoral responses^7,8^. Antibodies targeting *C. albicans* Sap2, cell wall proteins and Cht3 have shown to have protective effects in animal models due to enhanced opsonization^14,17^. Additionally, higher levels of TNF-α and IL-6 in BMDMs from DMCS-immunized suggest trained immunity, a response known to help in *C. albicans* control^9^.

Overall, from all our studies on DODAB:MO-based vaccines, the DMCS enhances immune responses by promoting antigen presentation, increasing antigen-specific IgG, Th1/Th17 cytokine, a favorable Th1/Th2 ratio, and BMDM pro-inflammatory responses. This improves survival and welfare in *C. albicans*-infected mice. In addition, the DODAB:MO liposomal formulation comprises components that are compatible with GMP (Good Manufacturing Practice) manufacturing processes, supporting its potential for future clinical translation.

## Methods

### Production and purification of recombinant Cht3 and Sap2

*Candida albicans* Cht3 was heterologous expressed in Pichia pastoris GS115 (Invitrogen) and purified as reported previously^15^. Purified Cht3 was concentrated using a PES 3KDa MWCO Pierce™ Protein Concentrator (Thermo Scientific) and washed three times with non-pyrogenic 25 mM HEPES buffer pH 7.5 (Thermo Scientific). *Candida albicans* ProSap2, consisting of both the propeptide and mature Sap2 sequences, was recombinantly expressed in *Escherichia coli* BL21(DE3) with the expression plasmid pET-22b(+) (Novagen) using procedures described previously^22^. The mature protein Sap2 was then purified using a previously described protocol^23^ before endotoxin removal with a Pierce™ High-Capacity Endotoxin Removal Spin Column used according to the manufacturer’s instructions. The protein solution was then washed three times with non-pyrogenic 20 mM Sodium Acetate Buffer, pH 5.2, using a PES 3 K MWCO Pierce™ Protein Concentrator.

Recombinant Cht3 and Sap2 purity were confirmed by SDS-PAGE with silver staining, and protein concentration determined with the Pierce™ BCA Protein Assay Kit (Thermo Scientific).

### Preparation of Cht3:Sap2-loaded DODAB:MO liposomes

2 mM DODAB:MO (at a molar ratio of 1:2) liposomes containing rCht3:rSap2 (DMCS) (at a final concentration of 50 µg/mL, i.e., 5 µg/mL Cht3 and 45 µg/mL Sap2) were produced using the thin lipid-film hydration method for liposomal preparation and the incubation method for protein incorporation as described previously^8^. For protein incorporation in the DMCS liposomes, two volumes of the liposomal formulation (at 4 mM) were mixed with one volume of Cht3 (at 20 µg/mL) and one volume of Sap2 (at 180 µg/mL), for final concentrations of 2 mM lipid, 5 µg/mL Cht3 and 45 µg/mL Sap2. Empty liposomes were also prepared mixing equal volumes of the liposomal formulation (at 4 mM) with 25 mM HEPES buffer pH 7.5, for a final concentration of 2 mM lipid.

### Characterization of DMCS liposomes

Liposomal formulation size, polydispersity, and surface charge were measured using a Malvern ZetaSizer Nano ZS at 25 °C. Morphology was analyzed by Cryo-SEM with a JEOL JSM 6301 F system. Samples were rapidly frozen, fractured, etched at −90 °C, sputter-coated with Au/Pd, and examined at −150 °C with a 15 kV acceleration voltage.

Cytotoxicity and cell activation were assessed using 100 µL of a 1 × 10^6^ cells/mL J774A.1 murine macrophages cultured in complete DMEM at 37 °C with 5% CO_2_. MTT and LDH assays evaluated cytotoxicity after 24 and 48 h, as previously described^8^, while TNF-α release was measured at 2, 6, and 24 h using an ELISA kit using the TNF alpha Mouse Uncoated ELISA Kit (Invitrogen). Macrophage morphology was also analyzed by microscopy after incubation with empty liposomes, DMCS, or free proteins for 1 h at 37 °C, 5% CO2, followed by fixation, gold sputter-coating, and SEM imaging with a Nova NanoSEM 200 microscope, FEI, at 10 kV. Macrophage morphology was analyzed by SEM after a 1 h incubation with empty liposomes, DMCS, or free proteins, followed by fixation, gold sputter-coating, and imaging with a Nova NanoSEM 200 microscope at 10 kV.

### In vivo analysis of immunization with DODAB:MO liposomes

#### Mouse husbandry

Female BALB/c mice, 8–10 weeks old, were purchased from Charles River (Barcelona, Spain) and kept under specific pathogen-free conditions at the Institute for Research and Innovation in Health (i3S), Porto, Portugal. After arrival, mice were allowed to acclimate for one week before being used. All procedures involving mice were performed according to the European Convention for the Protection of Vertebrate Animals used for Experimental and other Scientific Purposes (ETS 123), the 2010/63/EU directive, and Portuguese rules (DL 113/2013). Procedures were approved by the i3S institutional board responsible for animal welfare (ORBEA), and authorization to perform the experiments was issued by the competent national authority (Direção Geral de Alimentação e Veterinária) with the reference number 014036/2019-07-24.i3S.

#### Immunization procedures and blood and spleen collection

As determined in our previous studies^8,14,15^, each of three groups of eight BALB/c mice were injected subcutaneously, into the loose skin over the neck and shoulder area, every two weeks over a 4-week period with 200 µL of one of the following preparations: 25 mM HEPES Buffer pH 7.5, Free Cht3:Sap2 (5:45 µg/mL, total of 50 µg/mL), or DMCS. Mice were randomly distributed, and each cage contained animals from all the immunized groups. Three weeks after the final immunization, blood was collected by intracardiac injection from mice deeply anesthetized with isoflurane. Immediately after blood collection, the mice were euthanized by cervical displacement.

Spleens were harvested under aseptic conditions, and splenocytes were obtained by mechanical homogenization of the spleen in HBSS buffer (Sigma-Aldrich). Red blood cells were lysed using Ammonium–Chloride–Potassium Lysing Buffer (0.15 M Ammonium chloride, 0.01 M Potassium bicarbonate, 0.0001 M EDTA). Isolated splenocytes were washed and resuspended in RPMI-1640 medium (Sigma), supplemented with 10% heat-inactivated FBS, 4 mM glutamine, 10 mM HEPES, 1% penicillin/streptomycin, and 50 ng/mL β-mercaptoethanol (all from Sigma). To prepare serum, blood was allowed to clot and centrifuged at 12,000 × g, 15 min, 4 °C.

### “ex vivo” splenocyte proliferation

The CellTrace™ Violet Cell Proliferation Kit (Invitrogen) was used to assess the effects of Free rCht3:rSap2 and DMCS on cell proliferation. Splenocytes were prepared by centrifugation (300 × g, 10 min, 4 °C), resuspended in 1 mL PBS, and stained with an equal volume of 10 µM probe in PBS before being incubated in the dark at 37 °C in a humidified 5% CO_2_ atmosphere for 20 min. To neutralize unbound dye, cells were incubated with five volumes of complete RPMI medium on ice for 5 min. Following another centrifugation step, the cells were resuspended in RPMI and plated at a concentration of 1 × 10^5^ cells/well in a 96-well plate. Cell stimulation was performed for 6 days at 37 °C, 5% CO_2_, using 5 or 10 μg/mL Cht3:Sap2 at a ratio of 1:9. Unstimulated cells and cells treated with 1 μg/mL Anti-CD3 antibody were used as negative and positive controls for proliferation, respectively. After incubation, Violet-labeled cells were harvested by centrifugation, resuspended in FACS buffer, and stained with anti-mouse CD8 PE (clone 53-6.7, BioLegend) and anti-mouse CD4 FITC (clone RM4-5, BioLegend). After staining, the cells were washed, resuspended in FACS buffer, and analyzed by flow cytometry (BD FACSCanto II). Propidium Iodide (PI) was added to each sample before analysis to exclude dead cells. Data was processed using FlowJo (v10.9) analysis software.

### “ex vivo” splenocyte cytokine detection

To evaluate ex vivo cytokine production, 5 × 10^5^ spleen cells from animals immunized with either HEPES buffer, free Cht3:Sap2, or DMCS, were stimulated with Cht3:Sap2 (ratio of 1:9) at final concentrations of 5 or 10 μg/ml for 3 days at 37 °C and 5% CO_2_. Unstimulated cells and cells incubated with α-Anti-CD3 antibody (final concentration of 1 μg/ml) were used as negative and positive control for cytokine production, respectively. Cytokine concentrations of interferon (IFN)-γ, interleukin (IL)-4, IL-10, and IL-17 in the supernatants were measured using the Mouse Uncoated ELISA kit (Invitrogen).

### Semi-quantification of Cht3- and Sap2-specific antibody isotypes

Specific anti-Cht3 and anti-Sap2 immunoglobulins in the serum of immunized mice were quantified by the Enzyme-Linked ImmunoSorbent Assay (ELISA) as previously described^24^. Polystyrene microtiter plates (Nunc Maxisorp™) were coated with 5 µg/mL Cht3:Sap2 (proportion of 1:9), incubated overnight at 4 °C, and used for analysis of mice immunized with HEPES buffer, Free Cht3:Sap2, or DMCS. For this, all wells were blocked for 1 h at room temperature with 2% BSA in TST buffer (10 mM Tris, 150 mM NaCl, 0.05% Tween 20, pH 8.0) and serum samples were serially diluted in 1% BSA in TST buffer before being plated and incubated for 2 h at room temperature. After washing, alkaline phosphatase-conjugated goat anti-mouse IgG1, or IgG2a (Southern Biotechnology) was added and incubated for 1 h. Bound antibodies were detected by adding p-nitrophenyl phosphate (Sigma) and incubating for 30 min in the dark. The reaction was stopped with 0.1 M EDTA (pH 8.0), and absorbance was measured at 405 nm and 570 nm. Final values were calculated by subtracting the 570 nm reading from the 405 nm reading. Antibody titers were expressed as the highest dilution with absorbance twice that of the blank control (no serum).

### Analysis of cytokine profile of bone marrow derived macrophages

Femurs and tibias from immunized BALB/c mice were collected, and bone marrow cells were flushed with cold RPMI-1640. Cells were cultured in T25 flasks with complete RPMI supplemented with 20% L-929 cell line conditioned medium (LCCM) and incubated at 37 °C in a humidified atmosphere with 5% CO2. Half of the culture medium was replaced every 3 days. On day 10, Bone Marrow-Derived Macrophages (BMDMs) were detached, and purity of BMDM cultures was routinely assessed by flow cytometry. After FcγR blocking using purified anti-mouse CD16/CD32 (BD biosciences), cells were incubated with anti-mouse F4/80 Phycoerythrin (PE)-conjugate (clone BM8), anti-mouse I-A/I-E Peridinin Chlorophyll Protein Complex (PerCP)-conjugate (clone M5/114.15.2), anti-mouse CD45 FITC-conjugate (clone 30-F11) (all from BioLegend), and anti-mouse CD11b BV510-conjugate (clone M1/70; BD Biosciences) at previously determined optimal dilutions. Cells were analyzed in a FACSCanto II flow cytometer (BD Biosciences) using the FACSDiva software (BD Biosciences). Differentiation of BMDM consistently resulted in over 95% cells CD45+F4/80^+^CD11b^+^MHC class II^+^. BMDMs were plated at 1 × 105 cells/well in 96-well plates, and left to adhere overnight at 37 °C with 5% CO2. *C. albicans* SC5314 cells, grown overnight in Winge medium, were fixed with formalin:ethanol (1:9), washed, and resuspended in non-pyrogenic PBS. These cells were diluted in complete RPMI-1640 to 5 × 10^7^ cells/mL and incubated with BMDMs at a 1:5 multiplicity of infection (BMDM:*C. albicans*) for 24 h at 37 °C in a humidified atmosphere with 5% CO2. Untreated BMDMs incubated with medium alone served as negative controls. After incubation, supernatants were collected for TNF-α, IL-1β, and IL-6 quantification using Mouse Uncoated ELISA kits (Invitrogen), following the manufacturer’s protocol.

### Immunoprotection studies

To evaluate the protective effects of the different liposomal formulations, female BALB/c mice were immunized with the formulations/solutions as above and infected with *C. albicans* 124 A^14^. 10 animals were immunized with 25 mM HEPES buffer or empty 2 mM DODAB:MO liposomes; and groups of 8 animals were immunized with Free Cht3:Sap2 (5:45 µg/mL) or DMCS. *C. albicans* 124 A was grown for 14 h on Winge Agar plates at 30 °C, cells were scrapped with sterile loops, washed with non-pyrogenic sterile PBS, adjusted to 1 × 10^6^ cells/mL, and 100 µL used to infect each mouse by intravenous injection in the lateral tail vein. To confirm inoculum concentration, colony-forming units (CFU) counts on YPD agar plates (37 °C, 48 h) were carried out before and after infecting the animals. As expected, and previously reported^14^, infection progressed similarly in the HEPES buffer and Empty liposome groups (*P* = 0.5786), so these mice were included one Control group. To evaluate the progress of the hematogenously invasive candidiasis, the animal behavior, appearance, body weight, temperature (at the base of the sternum with a Bioseb 153 IRB infrared thermometer), hydration, respiratory movements were recorded daily and Welfare Score calculated (Fig. S4). Mice were euthanized by cervical displacement when achieving the humane endpoints or at the end of the experimental period.

### Statistical analysis

Data were analyzed with GraphPad Prism 9 software (GraphPad Software, Inc., La Jolla, CA), using analysis of variance (ANOVA) and the Bonferroni post hoc test to compare the mean values of different groups. Unless stated otherwise, results represent the average of three independent experiments with four replicates each, and differences were considered significant when the *P*-value was less than 0.05.

If your research involved human or animal participants, please identify the institutional review board and/or licensing committee that approved the experiments. Please also include a brief description of your informed consent procure if your experiments involved human participants.

## Supplementary information

Supplementary Information

SuplementaryTables

ARRIVE Compliance Questionnaire

## Data Availability

Raw data and supplementary information are available on the Open Science Framework, https://osf.io/vc5y6/?view_only=f84870a950644ad9b57ee07984320a04.
