# Supplementary material for: Pre-clinical evaluation of a divalent liposomal vaccine to control invasive candidiasis
Source: NPJ Vaccines. 2025 Jun 13;10:124. doi: 10.1038/s41541-025-01183-0 (PMC12166040; doi:10.1038/s41541-025-01183-0)
Supplement: Supplementary file 1 — Supplementary Information [file 41541_2025_1183_MOESM1_ESM.pdf]

Supplementary Information for

Pre-clinical evaluation of a divalent liposomal vaccine to control invasive candidiasis.

Augusto Costa-Barbosa[1,2], Maria Inês Pacheco[1,2], Andreia C. Gomes[1,2], Tony Collins[1,2], Manuel Vilanova[3,4], Célia Pais[1], Alexandra Correia [4] and Paula Sampaio[1,2]*

[1]Centre of Molecular and Environmental Biology (CBMA) /Aquatic Research Network (ARNET) Associate Laboratory, Universidade do Minho, Campus de Gualtar, 4710-057 Braga, Portugal

[2]Institute of Science and Innovation for Sustainability (IB-S), Universidade do Minho, Campus de Gualtar, 4710-057 Braga, Portugal

[3]i3S, Instituto de Investigação e Inovação em Saúde, Universidade do Porto, 4200-135 Porto, Portugal

[4]IBMC, Instituto de Biologia Molecular e Celular, Universidade do Porto, 4200-135 Porto, Portugal

* Paula Sampaio.

Email: psampaio@bio.uminho.pt

This PDF file includes:

Supplementary figures S1-S5

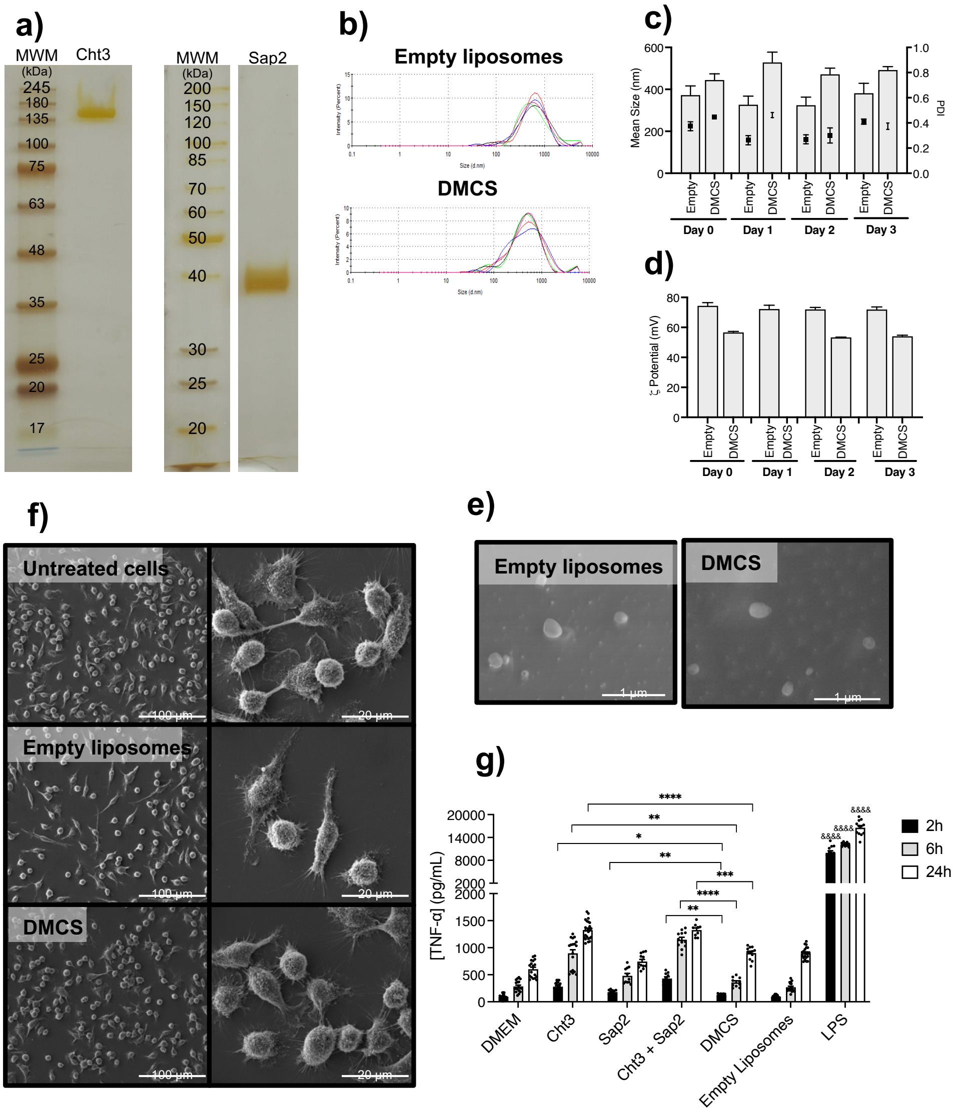

Figure S1 – **Liposomes characteristics. a)** Silver nitrate stained SDS-PAGE of purified recombinant Cht3 and Sap2. NZYColour Protein Marker II (left, nzytech) and PageRuler™ Unstained Protein Ladder (right, ThermoScientific) were used as Molecular Weight Marker (MWM). **b)** Size distribution of empty liposomes and DMCS (liposomes containing 1:9 Cht3:Sap2) as measured by dynamic light scattering (DLS). **c)** Particle size (bars) and polydispersity (squares) (PDI), and **d)** ζ potential of 2 mM DODAB:MO (1:2) empty liposomes and DMCS, measured at the time of protein encapsulation (day 0) and each day up to 3 days after preparation with storage at 4 ºC. **e)** Representative CryoSEM images of the morphology of the different DODAB:MO liposomal formulations. **f)** Representative SEM images of the morphology of untreated J774A.1 cells and after 1 hour incubation with: empty liposomes and DMCS. **g)** Quantification of TNF-α secretion by J774A.1 cells upon incubation for 2 h, 6 h and 24 h, with the following: DMEM, free Cht3, free Sap2, free Cht3:Sap2, DMCS, empty liposomes or LPS (1 µg/mL). Results are presented as the mean ± SEM of four independent experiments, each with three measurements. Significant differences between the free and encapsulated proteins are indicated by * above the bars (*P < 0.05; **P < 0.01; ***P < 0.001; ****P < 0.0001) and & indicates significant differences between LPS and all other conditions at each time point (&&&&, P < 0.0001).

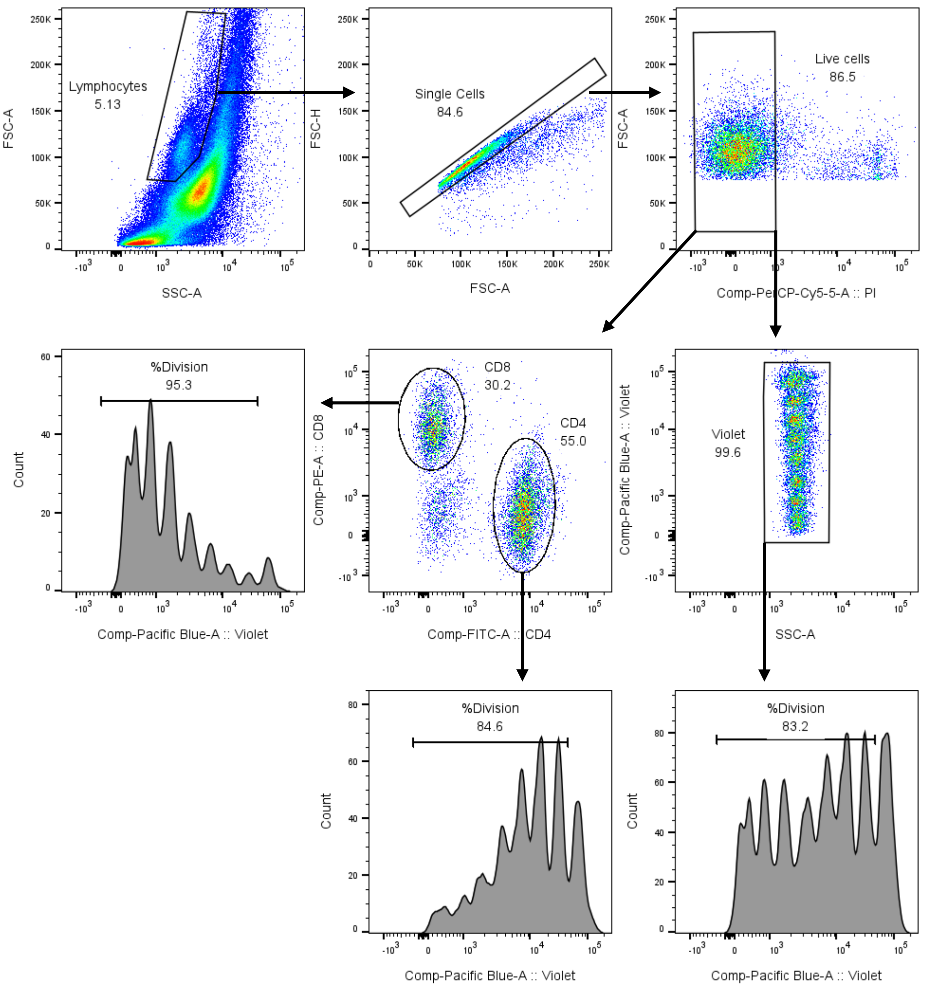

Figure S2 Gating strategy used to evaluate T cell proliferation by flow cytometry. Lymphocytes were selected based on FSC-A and SSC-A parameters and excluding cell debris; after doublet exclusion (single cells), live cells were selected based on propidium iodide (PI) incorporation; cells exhibiting violet staining were selected and represented in a histogram in which a marker defining the percentage of cells that divided at least once (% Division) is shown. Within total cells, CD4 and CD8 cell populations were defined according to the staining with specific antibodies. The percentage of cells that divided at least once are shown in the respective histograms. This is a representative example of cells from a mouse immunized with DMCS stained with Cell Trace Violet and stimulated with 1 ug/mL anti-CD3 mAb for 5 days.

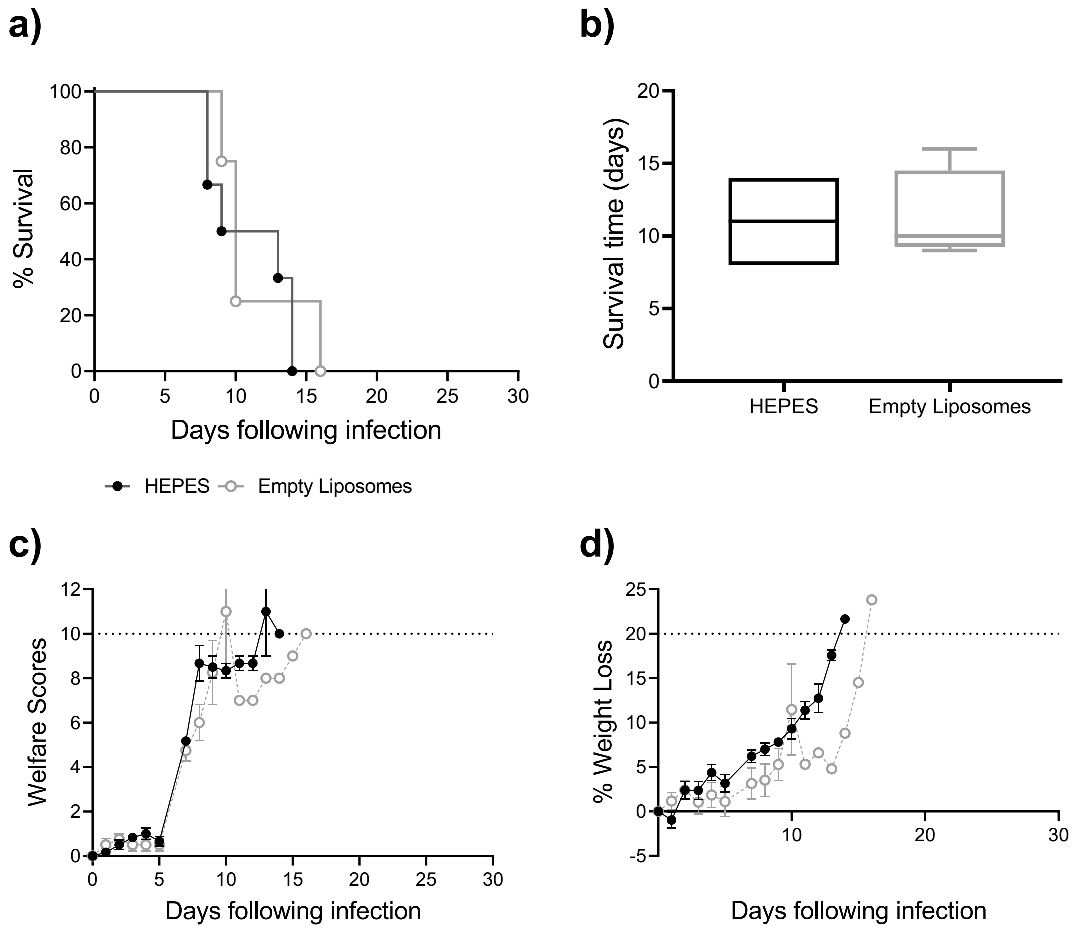

Figure S3 Survival of mice immunized with HEPES and with Empty liposomes after infection with *C. albicans*; **a)** Survival curve, **b)** Average survival time, **c)** Welfare scores and **d)** Percentage weight loss.

Score sheet for general procedures

Internal project ref: ____________________; Begin date: ___/___/_____; end date: ___/___/_____

Responsible(s) person(s): ____________________; ____________________ Group: __________________Extension: _________

Emergency contact (cell phone): ______________________ Strain: ______________________________

Parameter		Score	Date: __________________ Cage ID:_____________							
			Animal ID:	Animal ID:	Animal ID:	Animal ID:	Animal ID:	Animal ID:	Animal ID:	Animal ID:
**Body Weight**	**Initial weight (date):**									
	Current weight									
	No changes	0								
	BW loss 1-6%	1								
	BW loss 7-14%	2								
	BW loss 15-19%	3								
	**BW loss >20%**	**HEP**								
**Appearance**	Normal	0								
	General lack of grooming	1								
	Piloerection, ocular and nasal discharges, hunched up	2								
	Above and eyes half close	3								
**Behavior**	Normal	0								
	Minor changes or exaggerated response when provoked	1								
	Less mobile/isolated but alert	2								
	Restless or still, not alert	3								
**Hydration**	Normal	0								
	Abnormal skin test pinch	3								
**Respiratory movement**	Normal	0								
	Dyspnea	3								
	**Total score**	0-15								
**Notes/Comments:**										

0: Normal
1-4: Increase monitoring frequency (if one parameter scores 3 seek veterinary opinion).
5-9: Increase monitoring frequency. Reinforce feeding and hydration. Seek veterinary observation.
>10: Consider endpoint

Figure S4. Welfare assessment scoring sheet used for general procedures, including the criteria applied to determine humane endpoints and standardized survival endpoint during the infection study.

a) b)

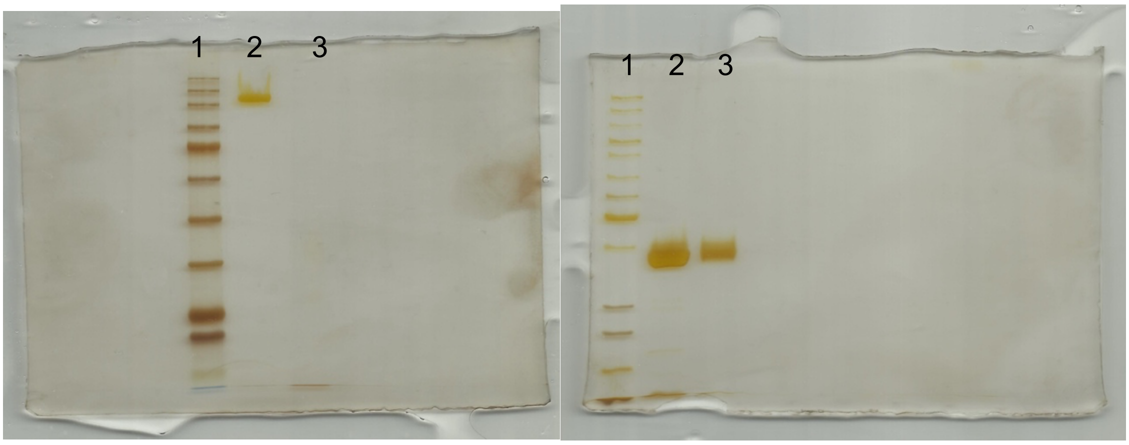

Figure S5- Uncropped and unprocessed images of the gels from figure S1a). Silver nitrate stained SDS-PAGE of purified recombinant Cht3 a) and Sap2 b). In the Cht3 gel, lane 1 shows the molecular weight marker (MWM), lane 2 displays the purified Cht3 protein after concentration by ultrafiltration (MWCO 3 kDa), and lane 3 represents the flow-through from the ultrafiltration process. In the Sap2 gel, lane 1 corresponds to the MWM, lane 2 shows the purified Sap2 protein prior to endotoxin removal, and lane 3 presents the purified Sap2 after passage through the endotoxin-removal column.
